# Non-Enzymatic Impedimetric Sensor Based on 3-Aminophenylboronic Acid Functionalized Screen-Printed Carbon Electrode for Highly Sensitive Glucose Detection

**DOI:** 10.3390/s19071686

**Published:** 2019-04-09

**Authors:** Ricardo Adriano Dorledo de Faria, Hassan Iden, Luiz Guilherme Dias Heneine, Tulio Matencio, Younès Messaddeq

**Affiliations:** 1 Department of Chemical Engineering, Universidade Federal de Minas Gerais (UFMG), Belo Horizonte, Minas Gerais 30270-901, Brazil; 2 Center for Optics, Photonics and Lasers (COPL), Université Laval, Quebec City, QC G1V 0A6, Canada; hassan.iden.1@ulaval.ca (H.I.); younes.messaddeq@copl.ulaval.ca (Y.M.); 3 CDN Isotopes, Montreal, QC H9R 1H1, Canada; 4 Department of Applied Immunology, Fundação Ezequiel Dias (FUNED), Belo Horizonte, Minas Gerais 30510-010, Brazil; luiz.heneine@funed.mg.gov.br; 5 Department of Chemistry, Universidade Federal de Minas Gerais (UFMG), Belo Horizonte, Minas Gerais 30270-901, Brazil; tmatencio@ufmg.br; 6 Institute of Chemistry, UNESP, Araraquara, São Paulo 14800-060, Brazil

**Keywords:** sensor, glucose, Electrochemical Impedance Spectroscopy, boronic acid

## Abstract

A highly sensitive glucose sensor was prepared by a one-step method using 3-aminophenyl boronic acid as a unit of recognition and a screen-printed carbon electrode (SPCE) as an electrochemical transducer. Scanning Electron Microscopy confirmed the success of the functionalization of the SPCE due to the presence of clusters of boronic acid distributed on the carbon surface. In agreement with the Electrochemical Impedance Spectroscopy (EIS) tests performed before and after the functionalization, Cyclic Voltammetry results indicated that the electroactivity of the electrode decreased 37.9% owing to the presence of the poly phenylboronic acid on the electrode surface. EIS revealed that the sensor was capable to selectively detect glucose at a broad range of concentrations (limit of detection of 8.53 × 10^−9^ M), not recognizing fructose and sucrose. The device presented a stable impedimetric response when immediately prepared but suffered the influence of the storage time and some interfering species (dopamine, NaCl and animal serum). The response time at optimized conditions was estimated to be equal to 4.0 ± 0.6 s.

## 1. Introduction

The detection of glucose has drawn significant attention due to the importance of this saccharide as a major dietary source of energy and its consequent impact on humans’ health. Glucose is the only carbohydrate present in the serum, and variations of its concentration can lead to hypoglycemia (in case of low concentration), diabetes and hypertension (in cases of high concentration) [1]. According to the World Health Organization, diabetes (whose criterion of diagnosis is the glucose concentration ≥ 7.0 mmol/L in the plasma) has been responsible for high levels of mortality worldwide [2]. 

Nonetheless, the importance of detecting glucose is not restricted to clinical applications. The necessity of improving quality control in the food industry has prompted the development of reliable analytical methods for the continuous monitoring of sugars aimed at increasing process efficiency and to prevent economic losses [3]. Many studies have been devoted to detecting glucose for various applications, such as beverage quality control [4,5], biological fuel cells [6,7] and environmental monitoring [8,9]. 

Optical and electrochemical transductions are the most widely studied methods for this purpose. In common, both of them are generally based on the conversion of the signal from the reaction between an enzyme (glucose oxidase is most widely used) and the glucose molecule, yielding metabolites that can change the color or electrochemical features of a certain matrix [10]. The hexokinase method, for example, consists of a series of chemical reactions involving glucose to the production of nicotinamide–adenine–dinucleotide-reduced (NADH), whose concentration is proportional to the absorbed light measured at the 340 nm wavelength [10]. 

As an advantage over the optical devices, electrochemical-based sensors have greater sensitivity. Among the electrochemical techniques, Electrochemical Impedance Spectroscopy (EIS) has attracted special attention not only due to its high sensitivity, but mainly because it allows the use of label-free and non-enzymatic methods [11,12]. In this technique, a sinusoidal potentiostatic perturbation is applied to an electrode under study, which is immersed in a conductive solution, generating a frequency-dependent AC current [13]. Thus, the frequency-dependent impedance is the complex ratio between the applied potential and the resultant current. The interpretation of impedance parameters (the inherent resistance, capacitance and/or inductance) can provide plenty of information on the interfacial phenomena that take place on the studied electrode, such as the recognition of an analyte of interest [14,15].

In the literature, there are several scientific articles devoted to the detection of glucose by enzymatic electrochemical sensors. Clark and Lyons [16] first reported the development of a device capable to detect glucose by means of the oxidation of the sugar mediated by the enzyme glucose oxidase (GOx) in the presence of oxygen and water producing hydrogen peroxide and gluconic acid. Since then, many studies have described methods to overwhelm the limitations of first-generation glucose sensors, for instance, the need for high operational potentials, the low activity of GOx or the interference of other sugars in the response of glucose dehydrogenase (an enzyme with higher activity than GOx) [17,18]. Although the majority of the commercial glucose sensors is based on enzymatic methods (which provides great sensitivity and selectivity), the use of enzymes possess some important limitations. The main disadvantages of the use of enzymes for bioapplications include the difficulty of immobilization due to their low stability (pH and temperature dependent activity), the high cost, low mobility and low mass-transfer rate [19,20,21].

Recently, several groups have directed great effort to developing glucose sensors with/without enzymes by using optical or electrochemical techniques [22,23,24], however, the enzymatic sensibility, the sensitivity of the device, selectivity, sophisticated fabrication and high cost still remain as important challenges to be overcome. 

In the present paper, we developed a 3-aminophenyl boronic acid-based sensor that detects glucose at low concentrations by using EIS as a transduction technique. This non-enzymatic sensor exhibited a low limit of detection and high selectivity towards glucose when exposed also to fructose and sucrose. The effect of interfering species commonly present in biological fluids was also examined, as well as the time of response of the sensor, its repeatability and reproducibility. Besides this, the use of screen-printed carbon electrode (SPCE) as a transducer substrate for this impedimetric sensor represents a key point of the presented technology since this material is well known to be cheap (with rapid and low-cost mass production), easily applied to point-of-care applications due to being portable and the easiness of functionalization [25,26,27].

## 2. Materials and Methods

### 2.1. Materials and Chemicals

Both 3-aminophenylboronic acid as sodium nitrate were purchased from J.T. Baker (Phillipsburg, NJ, USA), sulfuric acid 98% was purchased from Anachemia (Montreal, QC, Canada), potassium chloride and sodium chloride were purchased from Fisher Scientific (Brussels, Belgium), and D-(+)-glucose, D-(−)-fructose, sucrose, dopamine, potassium ferricyanide and potassium ferrocyanide were obtained from Sigma Aldrich (Oakville, ON, Canada). All chemicals were of analytical grade and the solutions were prepared using ultrapure deionized water (18 MΩ·cm resistivity). Bovine serum was provided by Fundação Ezequiel Dias (Belo Horizonte/MG, Brazil).

SPCE (RRPE1002C), consisting of carbon as working (with dimensions of 5 × 4 mm) and counter electrodes and Ag/AgCl as a reference electrode, were purchased from Pine Research.

### 2.2. Instrumentation and Apparatus

A Potentiostat VersaSTAT 3 from AMETEK Scientific Intruments (Mississauga, ON, Canada) was interfaced with the VersaStudio software. EIS was performed in an electrochemical cell containing 0.1M KCl as a supporting electrolyte and both K_3_[Fe(CN)_6_] and K_4_[Fe(CN)_6_] at 0.005 M as a redox probe couple.

Scanning Electron Microscopy (SEM) was performed using a Quanta 3D FEG microscope.

### 2.3. Functionalization and Characterization of the SPCE

Prior to the functionalization, the SPCE was submitted to a potential cycling from −2.5 to +2.5 V vs. Ag/AgCl in 0.05 M H_2_SO_4_ aqueous solution at 0.05 V.s^−1^ in order to remove its organic contaminants and to improve the surface electroactivity [28]. In our previous work, we demonstrated that this pre-treatment is crucial to enhancing the charge-transfer kinetics of the electrode by introducing hydroxyl groups on the surface and by exposing the graphite flakes edges from the carbon ink to the electrolyte [29].

**Figure 1:**
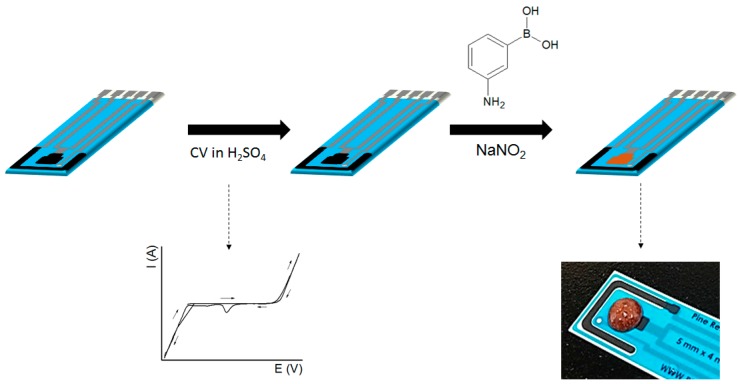
Functionalization steps to obtain the glucose sensor.

To the treated electrode, 20 μL of 0.4 M 3-aminophenylboronic acid and 20 μL of 0.4 M NaNO_2_ both diluted in 0.1 M H_2_SO_4_ were added to the working electrode. The reaction was left for 10 min, after which the electrode was washed exhaustively in a large volume of ultrapure water. The scheme presented in Figure 1 describes the steps to prepare the proposed glucose sensor.

EIS was performed to investigate the changes in the electrochemical behavior of the SPCE due to the functionalization. The test was carried out at an AC amplitude of 0.01 V around the open circuit potential (OCP), which was previously stabilized for 300 s, with a frequency range varying from 10,000 to 0.1 Hz. Five points per frequency decade were recorded.

### 2.4. Selective Detection of Glucose by EIS

The impedance of the functionalized sensor was determined by EIS with the same parameters previously presented to obtain a basal value. Afterwards, 60 μL of aqueous glucose solutions at 10^−8^, 10^−7^, 10^−6^, 10^−5^, 10^−4^, 10^−3^, 10^−2^, and 10^−1^ M were separately added to the working electrode, which was incubated for 300 s and carefully rinsed with a large volume of ultrapure water in order to remove the unbound and weakly adsorbed molecules from its surface. Finally, the electrode was immersed in the electrolyte and EIS was performed. This procedure was repeated for all glucose concentrations tested.

To assess the selectivity of the glucose sensor, its working electrode was exposed separately to aqueous solutions of both fructose and sucrose at the same glucose concentrations and EIS parameters.

The results were analyzed by modelling the EIS data to an appropriate electrical equivalent circuit in Zview software version 2.9b (Scribner and Associates). 

The effect of common interfering species on the performance of the sensor was investigated by EIS after exposing the device to 0.1 M NaCl, 0.02 M dopamine and bovine serum in the absence and presence of glucose at 10^−8^ M.

### 2.5. Evaluation of Sensor Stability

The chemical stability of the proposed glucose sensor was tested in terms of its repeatability according to the methodology described by Kannan and Rout [30], in which 100 consecutive EIS measurements were carried out. Additionally, the impedimetric response of the device was examined after 7, 14 and 21 days from its preparation.

## 3. Results and Discussion

### 3.1. Characterization of the Functionalized SPCE

**Figure 2:**
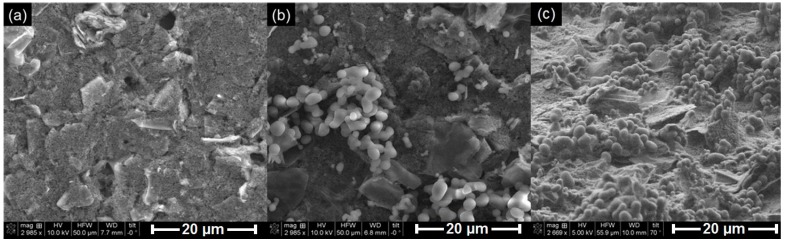
SEM images of the SPCE before (**a**) and after (**b**) functionalization with 3-aminophenylboronic acid and (**c**) lateral view of the functionalized electrode.

SEM images of the SPCE before and after the functionalization were recorded to verify the presence of the deposited boronic acid. Figure 2 shows that the bare electrode contained irregularly flaked graphite particles randomly distributed along the inhomogeneous surface of the carbon ink as also observed in the literature [31,32]. Following the incubation with the boronic acid, the transducer electrode was covered with clusters of circular shaped particles dispersed mainly around the graphite flakes.

**Figure 3:**
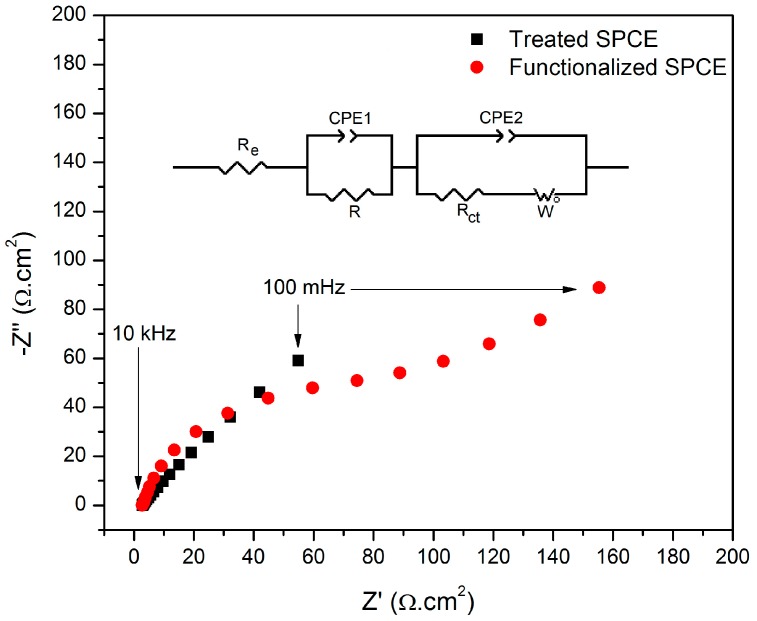
EIS monitoring of the SPCE before and after functionalization with 3-aminophenylboronic acid.

The functionalized electrode was also characterized by EIS. The Nyquist plot displayed in Figure 3 was interpreted by fitting the data to the electrical equivalent circuit presented in the figure. In the proposed circuit, “R_e_” is the electrolyte resistance and two times constants were considered to represent multiple charge transfer kinetics of the SPCE with a resistance “R”, the charge transfer resistance “R_ct_”, the constant phase elements “CPE1” and “CPE2” and the Warburg impedance “W”. The Warburg impedance is due to the diffusion of the ions Fe(CN)_6_^3-/4-^ from the bulk electrolyte to the electrode and it appears as a 45° straight line at low frequencies [33]. The CPE element represents a non-ideal capacitor and replaces the capacitance due to its roughness and heterogeneous surface [34]. The impedance associated to this parameter is described by Equation (1), where “Q” is a constant that describes the magnitude of CPE, “ω” is the angular frequency (ω = 2πf), “j” is an imaginary number (j = √−1) and “n” is an exponent related to the heterogeneity of the surface (0 < n < 1) [35]. Due to the complexity of the SPCE structure, consisting of the graphitic flakes and the carbon ink with different relaxation times, two pairs of time constants resistor/CPE were employed to fit the EIS data. The pair R/CPE1 arises from the graphitic phase which is more electroactive and, thus, possesses higher charge transfer kinetics, while the pair R_ct_/CPE2 corresponds to the more insulator carbon ink, whose mechanisms of charge transfer can be seen at lower frequencies followed by the diffusion of the electroactive species.

(1) $$CPE=\frac{1}{Q{\left(j\omega \right)}^{n}}$$

Considering the criterion of *χ*² < 10^−3^ as an indicative of the fitting quality [36], it was possible to ensure that the proposed equivalent circuit was suitable to model the experimental data.

The functionalization led to an increase of 96.156 Ω·cm² (1570.15%) in the R_ct_ of the SPCE due to the capability of the boronic acid to change the dielectric characteristics of the electrode, acting as a barrier to the electron transfer kinetics of the redox probe at the interface with the electrolyte [37].

The effect of the boronic acid to the electroactivity of the transducer matrix was evaluated by estimating the electroactive area of the SPCE before and after the functionalization by the Randles–Sevcik equation (Equation (2)). In the equation, “i_p_” is the peak current [A], “n” is the number of electrons involved in the redox reaction, “A” is the electroactive area [cm²], “C” and “D” are, respectively, the concentration [mol·cm^−3^] and the diffusion coefficient [cm²·s^−1^] of the redox species, and “*ν*” is the scan rate [V·s^−1^].

(2) ip=2.69×105×n32×A×C×D12×ν12

**Figure 4:**
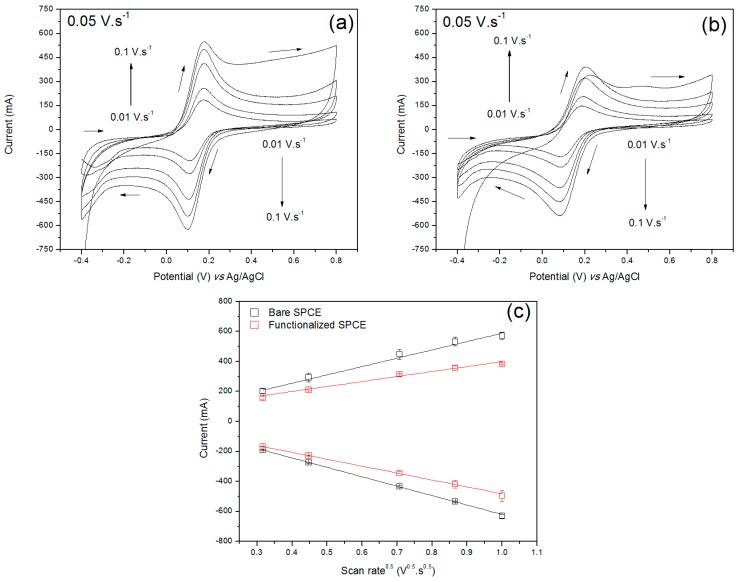
Cyclic voltammograms related to the bare (**a**) and functionalized (**b**) SPCE under different scan rates (0.1, 0.075, 0.05, 0.02 and 0.01 mV·s^−1^) in 0.1 M KCl + 5 mM Fe(CN)_6_^3-/4-^ and the relationship between the anodic/cathodic current peaks of the redox species at the electrodes and the square root of corresponding scan rates (**c**).

As seen in Figure 4, there was a linear trend between the square root of the scan rate led and both anodic and cathodic current peaks as a consequence of a reversible reaction mechanism limited by diffusion [38]. The electroactive area of the electrode, which possess a geometric area of 0.2 cm², was estimated as 0.4694 ± 0.0191 cm² (n = 3) before the functionalization. After the SPCE modification, there was a reduction of 37.9% of its electroactive area (A = 0.2915 ± 0.0083 cm²). This result corroborates the increase of R_ct_ from the Nyquist plot of Figure 3, demonstrating that the boronic acid was immobilized on the carbon surface as an insulating layer capable to hinder the interfacial charge transfer.

### 3.2. Detection of Glucose in Aqueous Solution by EIS

**Figure 5:**
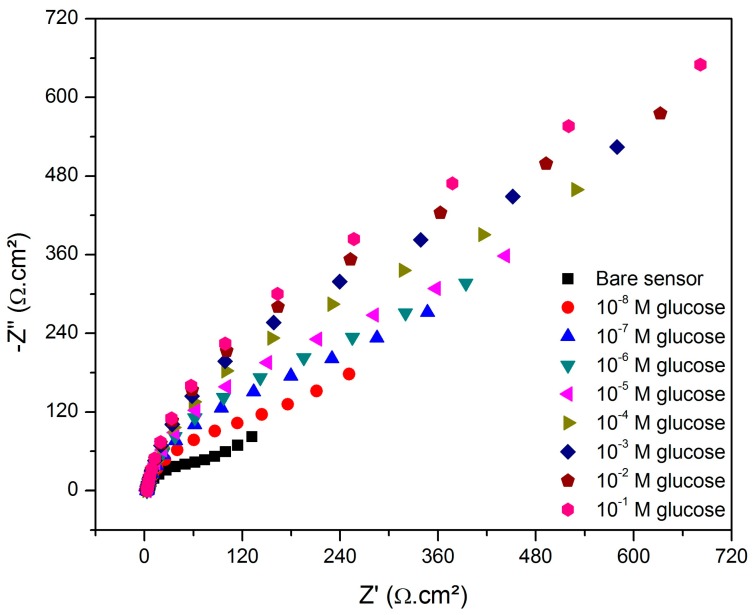
Nyquist plot of the glucose sensor exposed to the target analyte at various concentrations.

**Figure 6:**
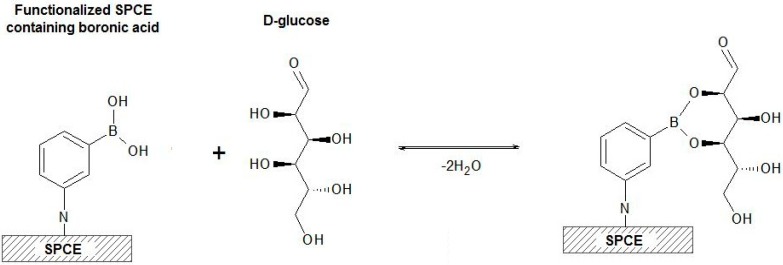
Scheme of the binding process between the boronic acid immobilized on the SPCE surface and the D-glucose molecule.

The functionalized SPCE was investigated toward the detection of glucose at various concentrations. As seen in Figure 5, the semicircle diameter increases with the increase of the target analyte concentration, which is indicative of the recognition capability of the sensor. This result was expected because, as shown in Figure 6, the boronic acid immobilized on the electrode surface interacts with the diols from glucose molecules forming a saccharide nonconductive layer that blocks the charge transfer between the ions [Fe(CN)_6_]^3-/4-^ on the interface with the electrolyte [37,38,39].

**Figure 7:**
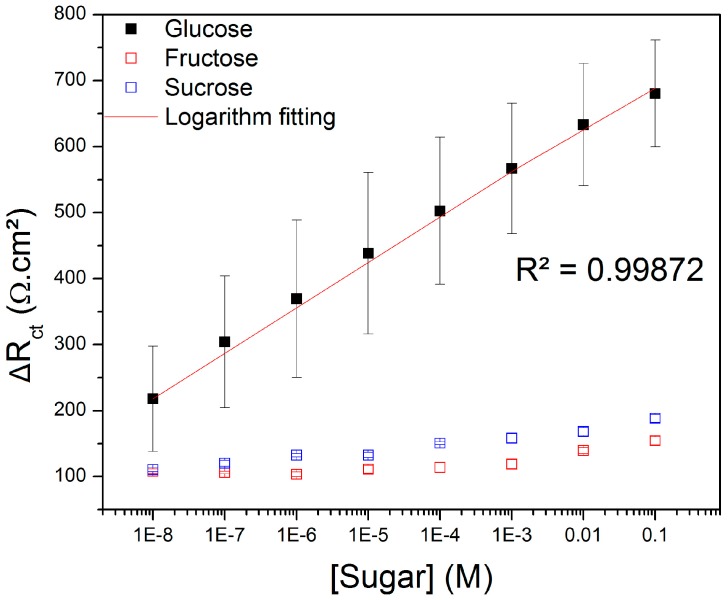
Variation of the charge transfer resistance (ΔR_ct_) of the glucose sensor exposed to glucose, fructose and sucrose at various concentrations.

The equivalent circuit previously described was employed to fit the experimental data related to the glucose detection. The variation of the R_ct_ from the modeling (ΔR_ct_) was used to plot the calibration curve shown in Figure 7. The ΔR_ct_ value represents the difference of R_ct_ from the sensor after and before its exposure to the different concentrated sugar solutions. It is evident the increase of ΔR_ct_ upon the exposition of the sensor to glucose at higher concentrations. A good linear correlation expressed by a logarithmic equation (Equation (3), R² = 0.99872) was obtained between the glucose concentration (from 10^−8^ to 10^−1^ M) and the ΔR_ct_. In this equation, y = ΔR_ct_ (Ω·cm²), x = log[glucose, M], a = 751.54 Ω·cm², b = −27.40 Ω·cm²·M^−1^ and c = −6.50 × 10^−9^ M. On the contrary, the exposure to fructose and sucrose did not increase the resistance of the sensor, but the R_ct_ randomly varied in the same concentration range. Since the mechanism of recognition of the proposed sensor was based on the bond between the diol groups from glucose and the boronic acid, some non-specificity could be observed in the impedimetric response of the sensor toward fructose and sucrose, because both possess the same target group. However, some authors have described different conditions that favor the selectivity of the interaction of just one of these saccharides by varying the pH, thermodynamic aspects, the nature of the electrolyte, etc. [40]. Wang et al. [37], for example, developed a gold-based impedimetric sensor using bis-boronic acid for the specific recognition of glucose (compared also to fructose, galactose and mannose).

(3) y=a−b×ln(x+c)

The sensitivity (S) of the sensor was estimated as the derivative of the logarithmic function between the output signal (ΔR_ct_) and the glucose concentration as expressed in Equation (4). As expected for a nonlinear plot (semi-logarithm), the sensitivity is a function of the analyte concentration. The lower was the glucose concentration, the higher was the sensitivity of the device was and its value is equal to 27.40/(x-6.50 × 10^−9^) in Ω·cm²·M^−1^.

(4) $$S=\frac{\partial y}{\partial x}=\frac{-b}{x+c}$$

The limit of detection (LOD) of the proposed sensor was estimated according to the 3SD/m criterion, in which “SD” is the standard deviation regarding the bare sensor and “m” is the slope of the linear plot of the calibration curve [41]. Based on this method, the LOD was equal to 8.53 × 10^−9^ M. As seen in Table 1, the proposed sensor was more sensitive than some others described in the literature by means of enzymatic and non-enzymatic devices based on optical and electrochemical techniques.

The low LOD of the proposed sensor indicates that this technology is a promising candidate for clinical applications. According to the literature, the level of glucose in the body of health patients varies from 4.4 to 6.6 mM [49], which is approximately 10^6^ times higher than the LOD of the proposed sensor. Furthermore, despite other transduction methods can achieve higher sensitivity, such as the amperometric device cited by Baghayeri et al. [48] that obtained LOD = 0.0003 µM, our proposed impedimetric sensor possesses incontestable advantages as the easiness of preparation by means of a single-step functionalization and the low cost of the SPCE.

### 3.3. Repeatability and Reproducibility Analysis

**Figure 8:**
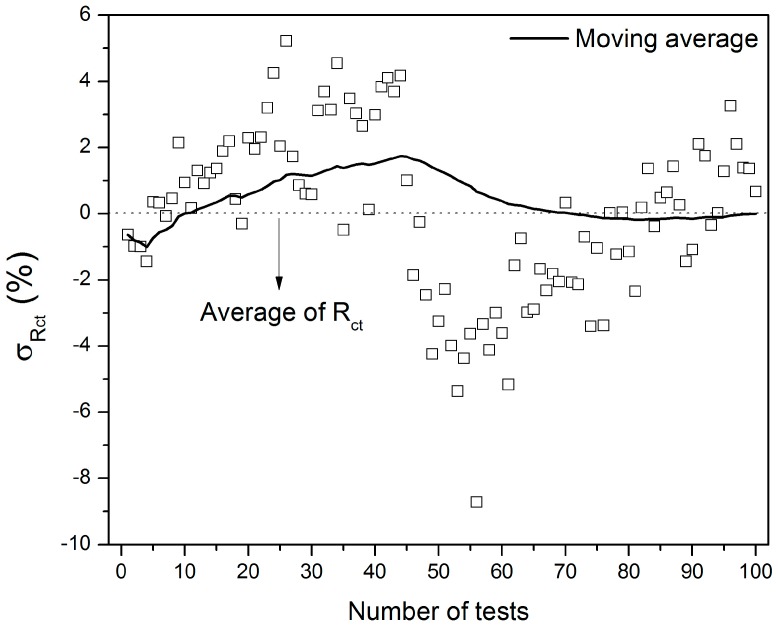
Relative residual errors referring to the impedance of the glucose sensor during 100 consecutive measurements.

The stability of the same sensor was assessed by examining the repeatability of its basal impedance over 100 consecutive measurements. The objective of this test was also to evaluate the robustness of the acquired data, ensuring that the oscillations on the R_ct_ values arising from the contact of the electrode with the electrolyte are not significant if compared to the variation caused by the analyte recognition. Figure 8 shows that the difference between the R_ct_ value from the first and the hundredth measurements was equal to 1.3%. Considering all the data, the R_ct_ varied by up to 8.7%, indicating that the developed sensor presents a significant repeatability.

**Figure 9:**
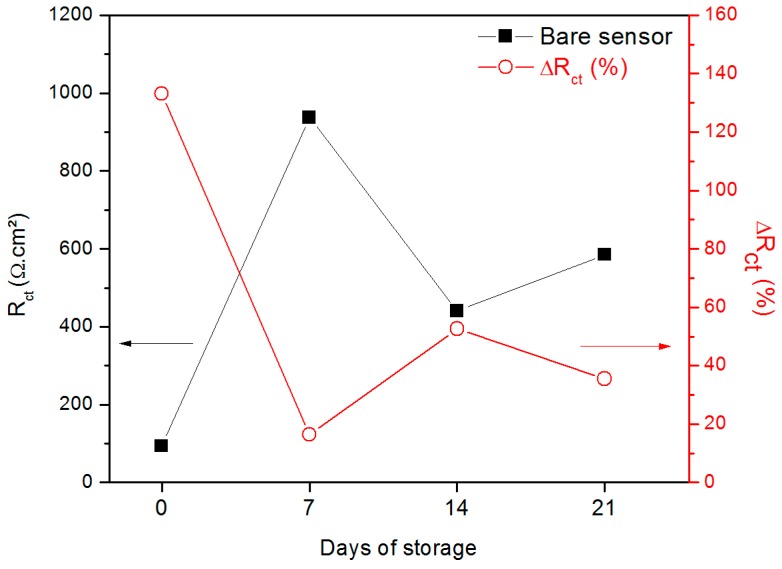
Variation of the R_ct_ of the sensor in function of the period of storage.

To evaluate the possibility of using this technology in practical applications, the stability of the sensor was examined by storing the device at room temperature in a dried and closed recipient during 21 days. EIS was performed every seven days after storage and the R_ct_ values were simulated by modelling the equivalent circuit. Next, the sensor was exposed to 10^−8^ M glucose in order to evaluate its sensitivity. The results presented in Figure 9 reveal that the impedance of the bare sensor increased during the period of storage with a maximum value in the seventh day, perhaps due to a sensor-to-sensor variation and not specifically to the time of incubation. The higher was the basal R_ct_ of the sensor, the lower was its capability to bind the target analyte was. The highest sensitivity was achieved with the fresh sensor, when the bare device presented an intrinsic R_ct_ equal to 93.5 Ω·cm² and, after the exposure to glucose at 10^−8^ M, experienced an increase of 133.2%. This fact suggests that the storage influenced the performance of the sensor, probably as a consequence of the redox reactions that spontaneously takes place on the carbon electrode, favoring the development of oxides on its surface. Thus, the formed insulator layer can act as a barrier to the charge transfer phenomenon and make the sensor unable to recognize the glucose molecules. In this sense, other storage conditions should be tested in order to maintain reproducible the performance of the device. 

### 3.4. Response Time

The response time (*τ*) can be defined as the time required by a sensor upon its exposure to a target analyte to yield a stable output signal [50]. Therefore, as the *τ* value is lower more appropriate the device is to be used for clinical diagnosis. In this work, to optimize the acquisition of the EIS data related to the detection of glucose, the values of the magnitude of impedance (|Z|) and the imaginary (Z”) and real (Z’) impedances were analyzed at five specific frequencies: 10^−1^, 10^0^, 10^1^, 10^2^ and 10^3^ Hz. This analysis allows determining the best set up to perform EIS at a single frequency.

**Figure 10:**
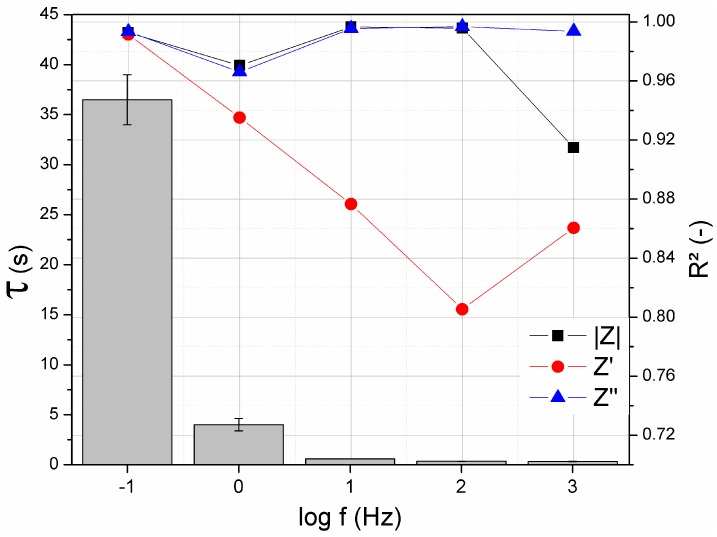
Relationship between the time to record |Z|, Z’ and Z” at 1000, 100, 10, 1 and 0.1 Hz and the respective correlation coefficient of these impedance parameters and the glucose concentration.

Figure 10 contains the results obtained by correlating the *τ* value in each frequency and the coefficient of linear regression (R²) of each impedance component due to the exposure of the sensor to glucose at various concentrations. Among the impedance components, Z” and |Z| presented the highest R² values indicating strong relationship between the concentration of glucose and these parameters in all the frequency range.

**Figure 11:**
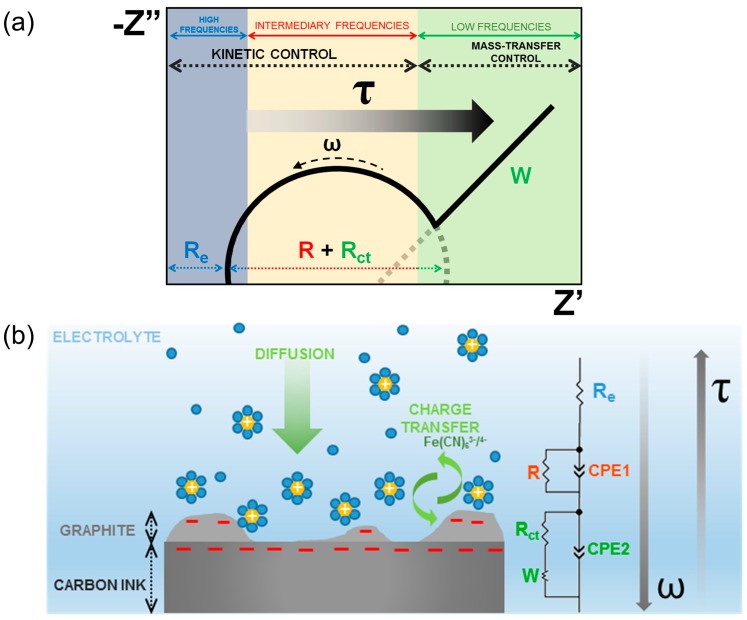
Scheme of the relationship among the time response and the impedance parameters as a function of the frequency in the Nyquist plot (**a**) as a consequence of the interfacial phenomena occurring between the SPCE and the electrolyte (**b**). In the illustration, the yellow spheres with the signal “+” correspond to the cations solvated by the molecules from the bulk solution (blue spheres) and the signal “−” on the different regions of the SPCE corresponds to the electrons electrostatically attracted toward the interfaces.

Obviously, the higher the frequency is, the lower is the time required to acquire the impedance data. It is well known that several phenomena interfere in the impedance spectrum at different frequency ranges, as illustrated in the scheme of Figure 11. At high frequencies, the impedance is strongly related to characteristics of the electrolyte, which is not representative of the analyte recognition. At very low frequencies, analysis can lead to the effect of diffusional mechanisms. For this reason, to avoid both non-representative effects of the detection of glucose, the analysis of EIS at 10^0^ Hz could provide the best response of the sensor considering the great R² values from both |Z| and Z’’ as well as the corresponding low *τ*, which was equal to 4.0 ± 0.6 s.

### 3.5. Investigation of the Effect of Dopamine, NaCl and Serum on the Performance of the Glucose Sensor

The capability of the sensor to detect glucose in solutions containing common interfering species for diabetes diagnosis was examined. Dopamine and NaCl are well known interferers studied in glucose detection [51,52] due to the possibility of charge transfer with electrochemical transducers. In this study, the effect of animal serum on the impedance of the sensor was also assessed in order to simulate the real environment (human blood) in which this device could be employed.

**Figure 12:**
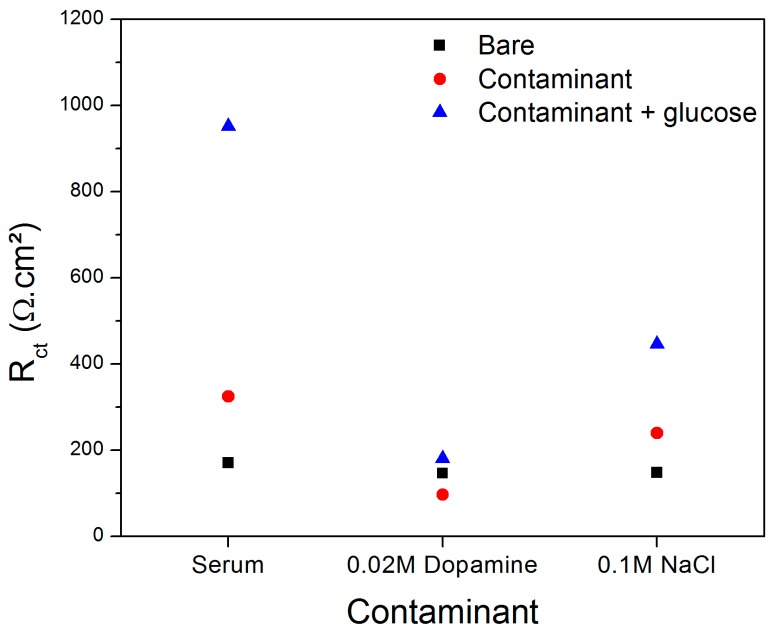
Variation of the R_ct_ of the glucose sensor in function of the interference of bovine serum, 0.02 M dopamine and 0.1 M in the absence and presence of glucose at 10^−8^ M.

Figure 12 shows the R_ct_ value of the bare sensor in absence/presence of each contaminant in a solution containing or not containing 10^−8^ M glucose. Despite the exposure of the sensor to the interfering species caused some variations on the impedance, a marked increase of R_ct_ occurred when the sensor was kept in contact with the solutions containing both glucose and interfering species. The presence of 10^−8^ M glucose in the solutions containing animal serum, dopamine and NaCl generated an increase of R_ct_ equal to 193.0%, 87.7% and 86.4%, respectively.

According to Yuan et al. [52], NaCl can cause the inactivation of some metal electrodes in the detection of glucose due to its poisoning effect on electrocatalysis. Rinaldi and Carballo [51] observed that human blood could interfere on the impedance of a glucose sensor but not significantly affected its sensitivity (slope of the calibration curve), obtaining a LOD equal to 0.37 mM. The exposure to dopamine was expected to decrease the impedance of the sensor since this molecule can interact to the conductive electrode in a reaction involving the exchange of two electrons and protons [53]. Thus, considering the performance of the impedimetric sensor to detect 10^−8^ M glucose in a medium containing the common interfering species NaCl, dopamine and the constituents of blood (herein simulated with animal serum), it is possible to infer that this device is a promising tool for monitoring the glucose levels in blood for diabetes diagnosis.

## 4. Conclusions

The fabrication of a non-enzymatic glucose sensor was reported based on the single-step attachment of 3-aminophenyl boronic acid on the surface of a SPCE. The changes in the electroactivity of the SPCE confirmed the success of the functionalization step, and SEM images revealed that the boronic acid molecules were incorporated as dispersed clusters on the surface of the electrode. EIS measurements were carried out for detecting glucose at various concentrations, and the sensor exhibited a broad linear range of detection with excellent selectivity to the target analyte, presenting a low signal to fructose and sucrose and a LOD equal to 8.53 × 10^−9^ M. The low LOD observed represents an important breakthrough in non-enzymatic glucose sensing since the use of enzymes possesses important limitations despite their high sensitivity. Further tests indicated that the sensor provided a stable signal and the detection could be performed at the single frequency of 1 Hz in order to minimize the response time. The storage time of the sensor was responsible for diminishing the capability of recognition at the studied experimental conditions. Finally, despite suffering some influence of dopamine, NaCl and animal serum as interfering species, the as-developed sensor is a promising tool for the detection of glucose, foremost due to its simple preparation, high sensitivity and the low cost of the SPCE.

## Figures and Tables

**Table 1:** Comparison of the performance of various enzymatic and non-enzymatic glucose sensors with the sensor based on SPCE containing 3-aminophenylboronic acid.

Use of Enzyme	Transducer Substrate/Unit of Recognition	LOD [µM]	Technique of Detection	Reference
No	SPCE containing 3-aminophenylboronic acid	0.0085	EIS	Present work
Yes	WS_2_ nanosheets	2.9	Spectrophotometry	[42]
Yes	chitosan-coated Fe3O4 NPs	0.43	Chemiluminescence	[43]
Yes	MoS_2_-based field-effect transistor	0.3	Measurement of the source-drain current	[44]
Yes	chitosan/-carrageenan polyelectrolyte complex	5	Square wave voltammetry	[45]
Yes	WS_2_ quantum dots	0.3	Fluorescence measurement	[1]
No	Ni NPs polyvinylpyrrolidone (PVP) stabilized graphene nanosheets (GNs) with chitosan (CS)	0.03	Chronoamperometry	[46]
No	NiO NPs electrodeposited on reduced graphene oxide–copper oxide nanocomposite bulk modified carbon ceramic electrode	2.63	Cyclic Voltammetry	[47]
No	Ag NPs on multiwall carbon nanotubes	0.0003	Amperometry	[48]

NPs = nanoparticles.
